# The Water Extract of *Ampelopsis grossedentata* Alleviates Oxidative Stress and Intestinal Inflammation

**DOI:** 10.3390/antiox12030547

**Published:** 2023-02-21

**Authors:** Zhaojie Wang, Qian Jiang, Pingping Li, Panpan Shi, Chao Liu, Wenmao Wang, Ke Huang, Yulong Yin, Peng Huang

**Affiliations:** 1 College of Animal Science and Technology, Hunan Agricultural University, Changsha 410128, China; 2 Hunan Qiankun Biological Technology Co., Ltd., Zhangjiajie 427000, China; 3 Hunan Key Laboratory of Traditional Chinese Veterinary Medicine, Hunan Agricultural University, Changsha 410128, China

**Keywords:** *Ampelopsis grossedentata*, Nrf2/Keap1 pathway, oxidative stress, anti-inflammatory, gut microbiota

## Abstract

Oxidative stress is recognized as a significant contributor to the development and progression of inflammation and disruptions in the balance of gut microflora, commonly referred to as intestinal dysbiosis. It is crucial that safe and effective antioxidant and anti-inflammatory agents are identified to address these conditions. *Ampelopsis grossedentata*, a natural plant abundant in flavonoids and primarily found in southern China, has demonstrated potent antioxidant properties. However, the extent to which flavonoids in *A. grossedentata* impact intestinal inflammation and alter the composition of the gut microbiome remains to be fully understood. The purpose of this study was to explore the potential benefits of using *A. grossedentata* as an antioxidant and anti-inflammatory agent in the context of intestinal inflammation, both in vitro and in vivo. We first conducted an initial comparison of the effects of dihydromyricetin (DMY), an alcohol extract of *A. grossedentata* (AEA, 82% total flavonoids), and a water extract of *A. grossedentata* (WEA, 57% total flavonoids) on the cell viability and intestinal barrier integrity of porcine epithelial cells IPEC-J2. Although the total flavonoid content is much lower in WEA than in AEA, the results show that they have similar effects. Subsequently, the antioxidant properties of WEA were compared with those of commonly utilized antioxidants in vitro. Lastly, the antioxidant and anti-inflammatory properties of WEA, as well as its impacts on gut microbiota, were evaluated in animal models, including mice and *Drosophila*. In summary, the results of our study indicate that WEA, due to its antioxidant properties, exhibits a protective effect on the intestinal barrier function in porcine epithelial cell line IPEC-J2. Additionally, WEA demonstrates a positive correlation with DPPH, ABTS radical scavenging rate, FRAP, and reducing power under in vitro settings. Furthermore, WEA was shown to effectively alleviate oxidative stress in animal models by reducing the levels of pro-inflammatory cytokines and increasing the antioxidant enzyme activity in the liver, as well as by activating the Nrf2 signaling pathway in the duodenum. Additionally, WEA was able to regulate gut microbiota, promoting the growth of beneficial bacteria and inhibiting harmful microbes, as well as extending the lifespan of *Drosophila*. Overall, these findings suggest that WEA may serve as a valuable dietary supplement for addressing oxidative stress and inflammation through its anti-inflammatory and prebiotic effects, which are conferred via the Nrf2/Keap1 pathway.

## 1. Introduction

Oxidative stress is caused by the overproduction of free radicals and reactive oxygen species (ROS), which leads to a decline in the body’s antioxidant defense ability [1]. Increased oxidative stress can cause cellular and tissue damage, resulting in the inability to resolve the inflammatory response, thereby leading to a chronic inflammatory state [2]. The status of oxidative balance plays an important role in maintaining the integrity of the intestinal mucosa through regulating its regeneration and repair [3]. Therefore, maintaining the structural integrity of the gut and reducing intestinal inflammation and oxidative stress play a key role in preserving the body’s health.

Plant secondary metabolites play an important role in human medicine and healthcare [4]. Therefore, many natural products have been widely used as antioxidant, antibacterial, and anti-inflammation agents [5]. Among the many available natural products, flavonoids are well-known antioxidant and anti-inflammatory substances that are now being used in the treatment and prevention of chronic diseases [6,7]. *Ampelopsis grossedentata* (Chinese name “Mei-Cha”), also called vine tea, is widely distributed in the southwest of China, and its young leaves have been used in beverages for centuries [8]. Previous studies have shown that *A. grossedentata* has a high content of total flavonoids and DMY, which are particularly enriched in dried leaves; as such, it is known as the “King of flavonoid-rich plants” [9]. Recently, it has been reported that *A. grossedentata* has a variety of pharmacological properties, and DMY has been identified as its principal medicinal substance, with antioxidant, anti-inflammatory, antibacterial, and hypoglycemic properties [10,11,12]. Previous studies have established that the activation of the nuclear factor-κB (NF-κB) signaling pathway is inseparable from the inflammatory response, and that the activation of the nuclear factor-E2-related factor 2 (Nrf2) signaling pathway can play an antioxidant role by promoting the expression of antioxidant enzymes in the body [13,14]. For example, studies have demonstrated that vine tea polyphenol activates the Nrf2-mediated expression of HO-1 and NQO1 [15]. However, details of the ability of the supplementation with *A. grossedentata* water extract to alleviate oxidative stress in vivo are not clear, and its target remains to be further confirmed [16]. Despite the fact that previous studies have demonstrated the ability of DMY to modulate the composition and interactions of gut microbiota, and the fact that the rat intestinal microbiota is responsible for the transformation of DMY into three specific metabolites [17], the underlying mechanisms and active ingredients responsible for the effects of berry tea on intestinal health and overall well-being remain unclear.

In the present study, we sought to clarify whether WEA alleviates oxidative stress and intestinal inflammation. First, the effects of DMY, AEA, and WEA on intestinal barrier function were investigated using an LPS-induced IPEC-J2 barrier damage model. Subsequently, the antioxidant performance of WEA was assessed via comparison with that of commonly used antioxidants in an in vitro environment. In addition, we also used an LPS-induced oxidative stress model in mice explore the molecular basis of the oxidative stress mitigation effect of WEA in depth, aiming to elucidate the mechanism by which it exerts this effect. Finally, we further explored the effects of WEA on the survival rate of *Drosophila melanogaster* using a paraquat-induced aging model.

## 2. Materials and Methods

### 2.1. Materials and Reagents

Dihydromyricetin (DMY, purity > 90%), an alcohol extract of *A. grossedentata* (AEA, total flavonoid content > 82%), and a water extract of *A. grossedentata* (WEA, total flavonoid content > 57%) were purchased from Hunan Qiankun Biological Technology Co. LTD., Zhangjiajie, China. Lipopolysaccharide (LPS), ferric-reducing antioxidant power (FRAP), tea polyphenols (TP), vitamin C (VC), N-acetylcysteine (NAC, anti-oxidant), and DEPC water were obtained from Beyotime Biotechnology. 2,2-Diphenyl-1-picrylhydrazyl (DPPH) and 2,2’-azino-bis (3-ethylbenzothiazoline-6-sulfonic acid) (ABTS) kits were purchased from Beijing Solarbio Science & Technology Co. Ltd. Methanol, potassium ferricyanide, trichloroacetic acid, ferric chloride, chloroform, isopropyl alcohol, and ethanol were provided by Sinopharm Chemical Reagent Co. Ltd., Shanghai, China. Paraquat was obtained from Chengdu Huaxia Chemical Reagent Co. Ltd. Interleukin 6 (IL-6), interleukin 1β (IL-1β), and tumor necrosis factor-alpha (TNF-α) ELISA kits were purchased from CHSABIO. Catalase (CAT) and the total antioxidant capability (T-AOC) kits were obtained from Beijing Boxbio Science & Technology Co. Ltd. IL-6, IL-1β, TNF-α, nuclear factor E2-related factor 2 (Nrf2), Kelch-like ECH-associated protein 1 (Keap1), and quinone oxidoreductase (NQO1) genes were purchased from Hunan Qingke Biotechnology Co. Ltd., Changsha, China. The TRIzol and RT Mix Kit with gDNA Clean for qPCR kits were provided by Accurate Biology. SYBR Green was obtained from Roche. Wild-type *Drosophila* w1118 was kindly provided by the Laboratory of Hunan Normal University. ICR mice and SPF chow were obtained from Changsha Tianqin Biotechnology Co. Ltd., Changsha, China. All chemicals were of analytically pure grade.

### 2.2. Cell Culture and Treatment

This study utilized porcine intestinal epithelial cells (IPEC-J2) obtained from the cell repository of Hunan Agricultural University. These cells were grown in Dulbecco’s modified Eagle’s medium containing 10% fetal bovine serum at 37 °C in a 5% CO_2_ atmosphere. The effects of WEA on the viability of IPEC-J2 cells were assessed through CCK-8 assays. The cells were treated with WEA and subsequently incubated for two hours at 37 °C, with the number of viable cells being determined by measuring absorbance at 450 nm using a microplate reader (TECAN, Infinite MPLEX, Mannedorf, Switzerland). The integrity of the IPEC-J2 cell monolayers was evaluated by assessing the transepithelial electrical resistance (TEER) and the permeation of fluorescein isothiocyanate-dextran 4 kDa (FITC-D4). The TEER was measured using an Epithelial Voltohmmeter (EVOM), while the FITC-D4 concentrations were determined by fluorimetry using a SpectraMax M2 microplate reader (TECAN, Infinite MPLEX, Mannedorf, Switzerland) at excitation and emission wavelengths of 485 and 530 nm, respectively.

### 2.3. In Vitro Antioxidant Activity

The measurement of the active ingredient content in WEA was undertaken using spectrophotometry. The total flavonoids, calculated as dihydromyricetin, were determined through a colorimetric assay utilizing aluminum trichloride and potassium acetate, with absorbance values measured at 294 nm. The polysaccharides, calculated as glucose, were determined through a colorimetric assay employing phenol and concentrated sulfuric acid, with absorbance values measured at 490 nm. The polyphenols, calculated as gallic acid, were determined through a colorimetric assay using forinol and sodium carbonate, with absorbance values recorded at 765 nm. Subsequently, standard curve equations were established, and the total flavonoids, polysaccharides, and polyphenols in WEA were calculated. Then, the antioxidant activity was evaluated using DPPH, ABTS, and FRAP free radical scavenging activity according to the manufacturer’s instructions. The absorbance was measured at 515, 405, and 505 nm with a microplate reader (TECAN, Infinite MPLEX, Mannedorf, Switzerland). The DPPH and ABTS radical scavenging ability was calculated as follows:

DPPH scavenging rate = (A0 − As)/A0 × 100%

ABTS scavenging rate = (A0 − As)/A0 × 100%

A0 is the absorbance of the control sample, and As represents the absorption of WEA or other standards.

The reducing power of WEA was determined via a method previously established by Oyaizu [18] with minor modifications. The samples WEA and TP were separately prepared at concentrations of 0.125, 0.25, 0.5, 1, 2, and 4 mg/mL and mixed with 300 μL potassium ferricyanide (0.01 g/mL). After 30 min of incubation at 50 °C in the dark, 300 μL trichloroacetic acid (0.1 g/mL) was added to stop the reaction; the tubes were then centrifuged at 3000 r/min for 15 min. Then, 300 μL of supernatant was mixed with 300 μL of distilled water and 100 μL aqueous ferric chloride (0.1 g/100 μL) solution. The absorbance was measured at 700 nm with a microplate reader (TECAN, Infinite MPLEX).

### 2.4. Animals and Drug Administration

**Figure 1:**
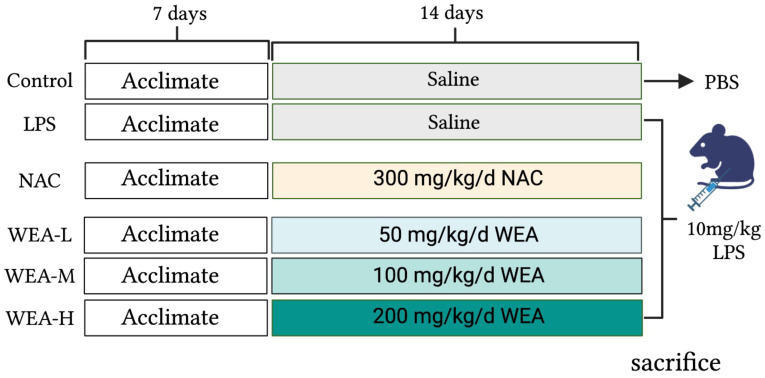
Study design. LPS, lipopolysaccharide; NAC, N-acetylcysteine.

The study was approved by the Animal Ethical Committee of Hunan Agricultural University. Forty-eight adult male ICR mice were maintained on a standard diet and water ad libitum. The mice were housed in a controlled environment with a consistent temperature, humidity, and light–dark cycle. After acclimatizing for one week, the mice were randomly divided into six groups (*n* = 8) (Figure 1), including the positive control (NAC) group, which was administered N-acetylcysteine (NAC) at a dose of 300 mg/kg/day [19]; the blank (control) and model (LPS) group, which was administered saline by oral gavage; and the low-, medium-, and high-dose WEA (WEA-L, WEA-M, WEA-H) groups were administered WEA at concentrations of 50, 100, and 200 mg/kg/day, respectively. To evaluate the safety and efficacy of WEA, fresh fecal samples were collected at the end of the experimental period and stored at −80 °C for subsequent analysis. On the 22nd day, the control group was intraperitoneally administered PBS, while the other groups, which had received LPS, NAC, and WEA, were administered lipopolysaccharide (LPS) at a dose of 10 mg/kg [20]. Subsequently, fresh fecal samples were collected from the model group. At the end of the treatment period, after a 6 h fast, the mice were weighed, and blood was collected from their eyes. The mice were then sacrificed, and the serum, liver, duodenum, jejunum, ileum, and intestinal contents were rapidly collected and stored at −80 °C for further biochemical analysis.

Subsequently, the concentrations of IL-6, IL-1β, and TNF-α in serum and T-AOC and CAT in the liver were analyzed utilizing appropriate diagnostic kits as per the manufacturer’s protocols. Total RNA was extracted from liver and duodenum tissue samples using TRIzol reagent and quantified using a Ultramicro ultraviolet spectrophotometer (Thermo Fisher, NanoDrop One, Shanghai, China). The extracted RNA was then converted to cDNA via reverse transcription utilizing the RT Mix Kit with gDNA Clean for qPCR kits. RT-qPCR was performed using SYBR Green, and the relative mRNA expression of the target genes was calculated with GAPDH as the internal control. The primer sequences are provided in Table S1.

### 2.5. Histological Analysis

The liver, duodenum, jejunum, and ileum fixed in 4% formaldehyde were used to determine morphology using hematoxylin–eosin staining. After dehydration, embedding, sectioning, and staining, the liver, duodenum, jejunum, and ileum were observed with a microscope. The villus height and crypt depth were measured using Case Viewer software 2.4.0.

### 2.6. Gut Microbiota Analysis

The microbial DNA present in the feces was extracted using an E.Z.N.A. Stool DNA Kit, and its purity was confirmed through 2% agarose gel electrophoresis. The pair-end library was constructed as per Illumina’s genomic DNA library preparation guidelines, using the NEXTFLEX Rapid DNASeq Kit. The compositions of the microbial communities present in the intestinal contents and feces samples were determined through 16S rDNA sequencing, carried out by Meiji Biological Co. Ltd. The amplicons were then sequenced on the Illumina MiSeq PE300 platform, utilizing the MiSeq Reagent Kit v3.

### 2.7. The Drosophila Lifespan Test

The *Drosophila* Lifespan Test of WEA adhered to a previous method with minor modifications [21,22,23]. For this test, *Drosophila* wild-type w1118 strain was utilized to evaluate the lifespan. The experiments were conducted under controlled conditions at 25 °C and a 12 h light–dark cycle. A sample of 180 healthy *Drosophila* individuals was selected and divided into four groups: the control group, the 0.1 mg/mL VC group, and groups treated with WEA at doses of 0.05 and 0.2 mg/kg. The experimental design was replicated in triplicate, with each group consisting of 15 *Drosophila* individuals per food vial. The food vials were replaced every other day. After a period of three days, the *Drosophila* were treated with 6 mM paraquat. The survival of the *Drosophila* was subsequently monitored and recorded at 0, 12, 15, 18, 21, 24, and 36 h. The lifespan curves were plotted for each group, and statistical analyses were performed to determine the mean lifespan, 50% survival rate, and maximum lifespan of the *Drosophila*. The maximum lifespan was calculated as the mean lifespan of the longest surviving 10% of *Drosophila* individuals.

### 2.8. Statistical Analysis

All experimental data were expressed as the mean ± standard error of the mean (SEM). The data were analyzed using IBM SPSS Statistics 23 and GraphPad Prism 9.0 software. Differences in the experimental groups were determined using the one-way analysis of variance (ANOVA). *p* < 0.05 was deemed a significant difference.

## 3. Results

### 3.1. Effect of DMY, AEA, and WEA on IPEC-J2 in LPS Model of Inflammatory Injury

**Figure 2:**
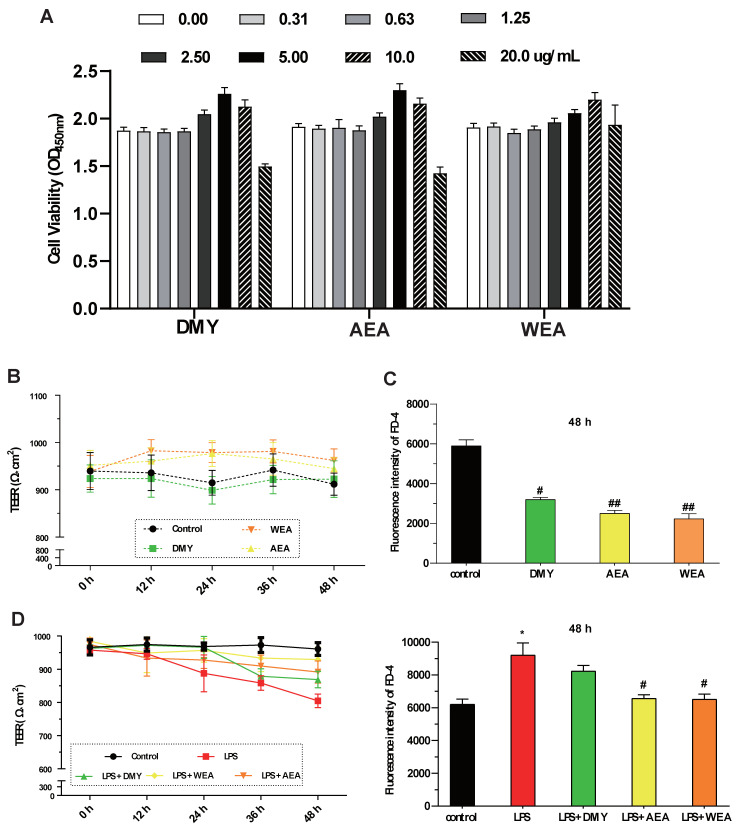
Effects of DMY, AEA, and WEA on the intestinal barrier function, as determined based on IPEC-J2 cells. Effects of DMY, AEA, and WEA on IPEC-J2 (**A**) cell viability, (**B**) transmembrane resistance (TEER), (**C**) permeability (FD-4 leakage), and (**D**) TEER value (**D**, left) and FD-4 leakage (**D**, right) when using LPS to simulate inflammatory injury in cell monolayers. All values are expressed as mean ± SEM. Compared with control * *p* < 0.05. Compared with LPS, ^#^ *p* < 0.05, ^##^ *p* < 0.01. DMY = 5 μg/mL dihydromyricetin ( purity > 90%); AEA= 5 μg/mL alcohol extract of *A. grossedentata* (total flavonoid content > 82%); WEA = 10 μg/mL water extract of *A. grossedentata* (total flavonoid content > 57%); LPS = 5 μg/mL lipopolysaccharide; LPS + DMY = 5 μg/mL dihydromyricetin + 5 μg/mL lipopolysaccharide; LPS + AEA = 5 μg/mL alcohol extract of *A. grossedentata* + 5 μg/mL lipopolysaccharide; LPS + WEA = 10 μg/mL water extract of *A. grossedentata* + 5 μg/mL lipopolysaccharide.

- DMY (5.0 μg/mL), AEA (5.0 μg/mL), WEA (10.0 μg/mL) significantly increased the IPEC-J2 cell viability. However, DMY, AEA, and WEA at a high concentration (20.0 μg/mL) all decreased cell viability (Figure 2A). AEA (5.0 μg/mL) and WEA (10 μg/mL) tended to increase the transmembrane resistance (intestinal integrity) of the intestinal barrier in vitro (Figure 2B). Meanwhile, DMY (5.0 μg/mL) (*p* < 0.05), AEA (5.0 μg/mL) (*p* < 0.01), and WEA (10 μg/mL) (*p* < 0.01) significantly reduced the permeability of the IPEC-J2 cells used to mimic the intestinal epithelial barrier (Figure 2C). AEA and WEA alleviated the damage caused by LPS (5 μg/mL) in the intestinal barrier (*p* < 0.05; Figure 2D), with similar effects.

### 3.2. Investigation of the Chemical Composition and In Vitro Antioxidant Properties of WEA

**Figure 3:**
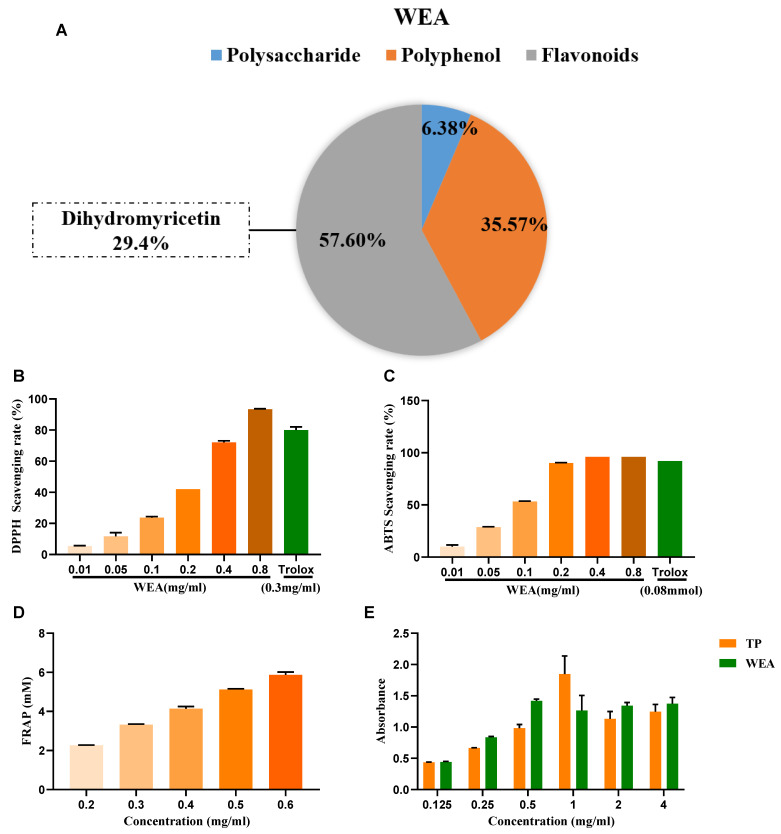
Antioxidant ability of WEA. (**A**) The main active ingredients in WEA. (**B**) Scavenging activities of WEA on DPPH radical, (**C**) ABTS radical, (**D**) FRAP, and (**E**) reducing power in vitro. Trolox and TP were used as positive controls. DPPH, 2,2-diphenyl-1-picrylhydrazy; ABTS, 2,2’-azino-bis(3-ethylbenzthiazoline-6-sulfonic) acid; FRAP, ferric-reducing antioxidant power; TP, tea polyphenols; WEA, water extract of *A. grossedentata*.

- The active compounds present in WEA were identified through chemical analysis, revealing that WEA comprises 57.6% flavonoids, 35.37% polyphenols, and 6.38% polysaccharides. Among the flavonoids, 29.4% were identified as dihydromyricetin (Figure 3A). Then, the antioxidant activity of WAE in vitro was evaluated by 1,1-diphenyl-2-picrylhydrazyl (DPPH), 3-ethyl-benzothiazoline-6-sulfonic acid (ABTS), ferric-reducing antioxidant power (FRAP), and reducing power assays (Figure 3B–E). The DPPH free radical is a very stable nitrogen-centered free radical and one of the most important indicators of antioxidant capacity [24]. Therefore, DPPH is widely used in the research into antioxidant foods, health products, and medicines. Our results show that WEA (0.2–0.8 mg/mL) had good DPPH free radical scavenging ability, and this increased with increases in component concentration, showing a concentration-dependent effect (Figure 3B). The ABTS cationic free radical is a colored cationic free radical, and it is also one of the more effective means of evaluating the free radical scavenging ability of protein hydrolysate [25]. The radical scavenging rate of ABTS in WEA (0.2–0.8 mg/mL) was near 96% (Figure 3C), similar to that of Trolox. Synchronously, the FRAP values of WEA reached 12.43 mM at the concentration of 0.4 mg/mL (Figure 3D). Moreover, WEA had a certain reducing power, which also showed dosage dependence (Figure 3E). In conclusion, the antioxidant activities of WEA were positively correlated with the concentrations of the samples.

### 3.3. Effect of WEA on the Level of Serum Inflammatory Cytokines

**Figure 4:**
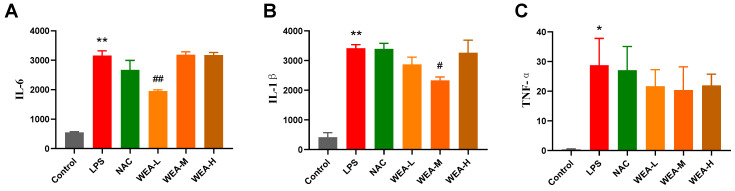
Serum inflammatory factor levels in mice. (**A**) IL-6, (**B**) IL-1β, and (**C**) TNF-α levels in each group of mice were quantified by ELISA. All values are expressed as mean ± SEM. Compared with Control * *p* < 0.05, ** *p* < 0.01. Compared with LPS, ^#^ *p* < 0.05, ^##^ *p* < 0.01. IL-6, interleukin 6; IL-1β, interleukin 1β; TNF-α, tumor necrosis factor alpha. LPS = 10 mg/kg lipopolysaccharide; NAC = 300 mg/kg N-acetylcysteine + 10 mg/kg lipopolysaccharide; WEA-L = 50 mg/kg water extract of *A. grossedentata* + 10 mg/kg lipopolysaccharide; WEA-M = 100 mg/kg water extract of *A. grossedentata* + 10 mg/kg lipopolysaccharide; WEA-H = 200 mg/kg water extract of *A. grossedentata* + 10 mg/kg lipopolysaccharide.

- The above in vitro tests show that WEA had strong antioxidant activity, so we first studied its antioxidant mechanisms in mice. We found that the serum levels of IL-6 and IL-1β in the LPS group were significantly higher than in the control group (*p* < 0.01; Figure 4A,B), indicating the successful establishment of the LPS model in the current study. Additionally, the serum IL1–6 (*p* < 0.01; Figure 4A) and IL1-β (*p* < 0.05; Figure 4B) levels were significantly lower in the WEA-L and WEA-M groups than in the LPS group. Although the serum TNF-α level was also lower in the WEA groups, these differences were not significant (*p* > 0.05; Figure 4C). However, a very minimal dose-dependent effect of WEA was observed on the three inflammatory cytokines, which may be due to the individual differences between mice.

### 3.4. WEA Alleviates LPS-Induced Injury by Inhibiting Liver Oxidative Stress and Inflammatory Cytokines

**Figure 5:**
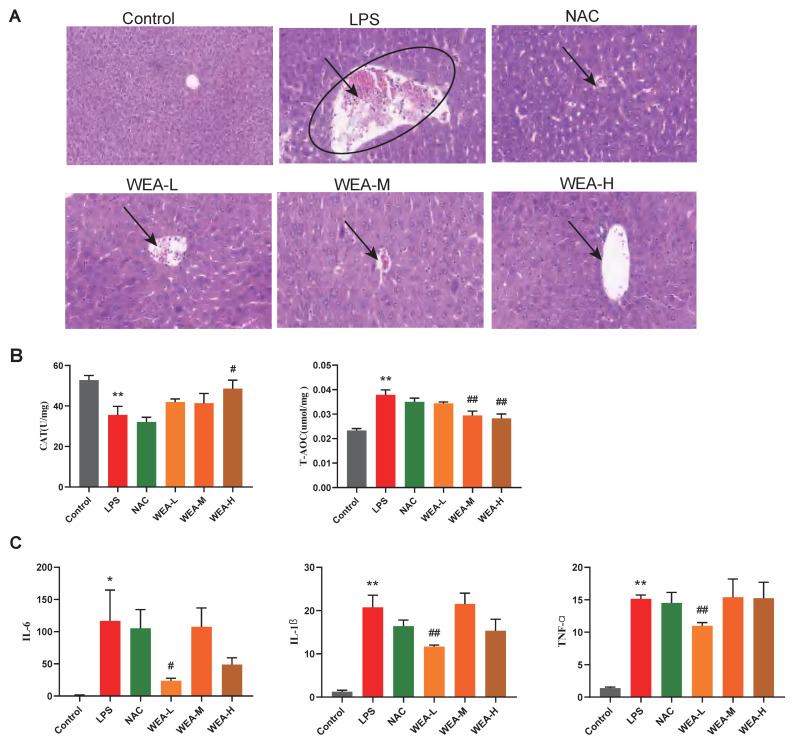
Liver morphology, antioxidant capacity, and inflammatory factor gene expression in mice. (**A**) Representative images of liver histology, all captured at 200× magnification. (**B**) CAT and T-AOC activities in the livers of mice treated with different WEA concentrations. (**C**) IL-6, IL-1β, and TNF-α expression in the livers of mice treated with different WEA concentrations. All values are expressed as the mean ± SEM. Compared with Control * *p* < 0.05, ** *p* < 0.01. Compared with LPS, ^#^ *p* < 0.05, ^##^ *p* < 0.01. CAT, catalase; T-AOC, total antioxidant capacity; IL-6, interleukin 6; IL-1β, interleukin 1β; TNF-α, tumor necrosis factor alpha. LPS = 10 mg/kg lipopolysaccharide; NAC = 300 mg/kg N-acetylcysteine + 10 mg/kg lipopolysaccharide; WEA-L = 50 mg/kg water extract of *A. grossedentata* + 10 mg/kg lipopolysaccharide; WEA-M = 100 mg/kg water extract of *A. grossedentata* + 10 mg/kg lipopolysaccharide; WEA-H = 200 mg/kg water extract of *A. grossedentata* + 10 mg/kg lipopolysaccharide.

- To investigate the reparative effects of WEA on the liver, we collected liver samples from mice for histopathological analyses. Figure 5A shows that the liver lesions of the LPS group displayed necrosis, vacuolar degeneration, and the infiltration of the inflammatory cells, which indicate serious hepatocellular damage. However, the WEA-L and WEA-M groups showed less damage compared with the LPS-induced mice, and the WEA-H group showed only mild vacuolar degeneration and infiltration of the inflammatory cells, the effect of which was similar to that in the control and NAC groups. These results indicate that WEA might attenuate the liver injury of LPS-induced mice.
- Then, to estimate the mechanisms by which WEA alleviates the liver injury caused by LPS, we examined the activity levels of CAT and T-AOC as well as the expression of inflammatory cytokines in the liver. In this study, LPS reduced the CAT levels and increased the T-AOC level compared with the control group (*p* < 0.01; Figure 5B). On the other hand, WEA-H increased CAT (*p* < 0.05; Figure 5B) levels and WEA-M and WEA-H reduced the T-AOC (*p* < 0.01; Figure 5B) levels. In contrast, T-AOC levels were higher in the LPS group, which may be due to the fact that oxidative stress was more severe in the LPS group, resulting in the greater production of antioxidant substances and antioxidant enzymes to resist the stress response. The real-time PCR results indicate that the pro-inflammatory cytokines, including IL-6 (*p* < 0.05), IL-1β (*p* < 0.01), and TNF-α (*p* < 0.01), were significantly increased in the LPS group compared with the control group (Figure 5C). However, WEA-L reduced the levels of IL-6 (*p* < 0.05; Figure 5C), IL-1β (*p* < 0.01; Figure 5C), and TNF-α (*p* < 0.01; Figure 5C). Taken together, these indicate that WEA is able to alleviate the abnormal oxidative stress and pro-inflammatory cytokine secretion induced by LPS.

### 3.5. WEA Promotes Nrf2 Expression in the Duodenum and Activates the Nrf2/Keap1 Pathway in Mice

**Figure 6:**
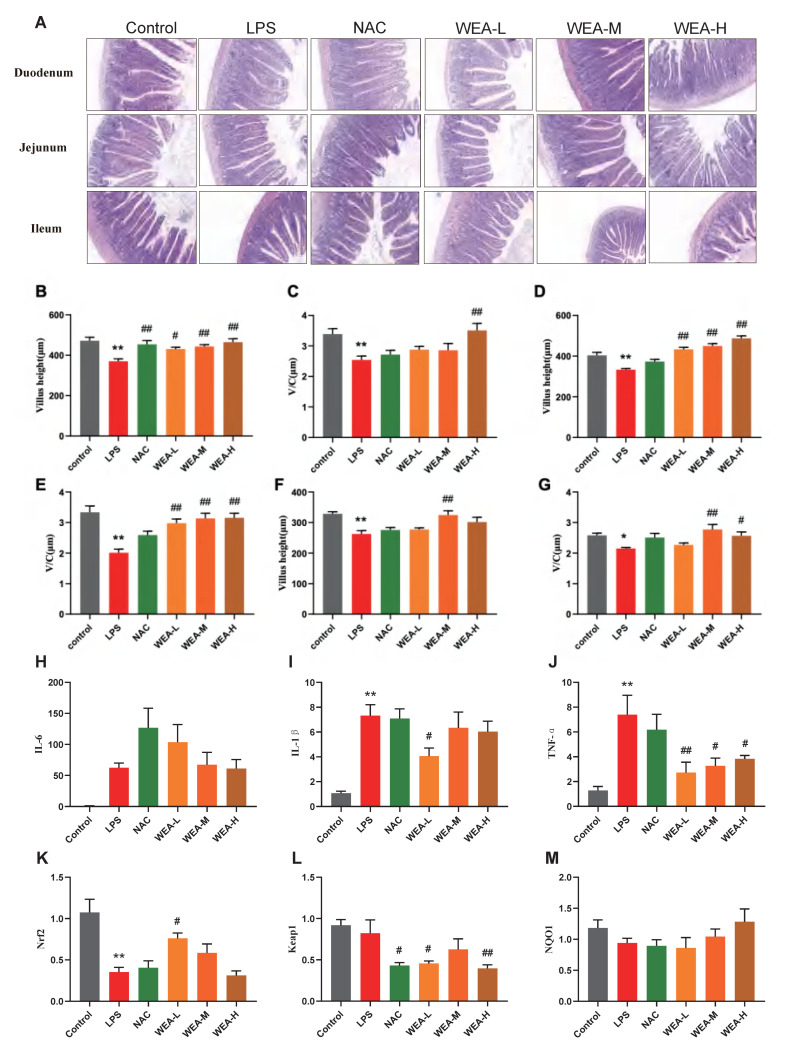
Intestinal morphology of mice and gene expression of pro-inflammatory and Nrf2-related genes of duodenum in mice. (**A**) Representative images of duodenum, jejunum, and ileum histology; all captured at 200× magnification. (**B**) Duodenum villus height and (**C**) ratio of villus height to crypt depth; (**D**) jejunum villus height and (**E**) ratio of villus height to crypt depth; (**F**) ileum villus height and (**G**) ratio of villus height to crypt depth. (**H**) IL-6, (**I**) IL-1β, and (**J**) TNF-α expressions in the duodenum of mice treated with different WEA concentrations. (**K**) Nrf2, (**L**) Keap1, and (**M**) NQO1 expression in the duodenum of mice treated with different WEA concentrations. Values are shown as mean ± SEM. Compared with control * *p* < 0.05, ** *p* < 0.01. Compared with LPS, ^#^ *p* < 0.05, ^##^ *p* < 0.01. IL-6, interleukin 6; IL-1β, interleukin 1β; TNF-α, tumor necrosis factor alpha; Nrf2, nuclear factor E2-related factor 2; Keap1, Kelch-like ECH-associated protein 1; NQO1, quinone oxidoreductase. LPS = 10 mg/kg lipopolysaccharide; NAC = 300 mg/kg N-acetylcysteine + 10 mg/kg lipopolysaccharide; WEA-L = 50 mg/kg water extract of *A. grossedentata* + 10 mg/kg lipopolysaccharide; WEA-M = 100 mg/kg water extract of *A. grossedentata* + 10 mg/kg lipopolysaccharide; WEA−H = 200 mg/kg water extract of *A. grossedentata* + 10 mg/kg lipopolysaccharide.

- To assess the therapeutic effects of WEA on intestinal injury, we collected duodenum, jejunum, and ileum samples from mice for histopathological analysis (Figure 6A). The morphological analysis revealed that there was less shedding of intestinal villi, which were more integrated, in the groups treated with NAC and WEA compared with the LPS-induced group, signifying the potential use of WEA in ameliorating the impaired intestinal morphology. Furthermore, the morphological analysis revealed that the WEA-L (*p* < 0.05), NAC (*p* < 0.01), WEA-M (*p* < 0.01), and WEA-H (*p* < 0.01) treatments led to a statistically significant increase in the height of villi within the duodenum (Figure 6B). The analysis revealed that the WEA-H treatment resulted in a higher ratio of villus height to crypt depth within the duodenum (*p* < 0.01; Figure 6C). Similar results were observed in the jejunum tissue, where WEA-L, WEA-M, and WEA-H treatments restored the villus height (*p* < 0.01; Figure 5D) and the ratio of villus height to crypt depth (*p* < 0.01; Figure 6E). In the ileum tissue, the WEA-M treatment was found to improve the villus height (*p* < 0.01; Figure 5F). Moreover, the study results indicate that the WEA-M (*p* < 0.01) and WEA-H (*p* < 0.05) treatments resulted in a notable increase in the ratio of villus height to crypt depth, as demonstrated by the data presented in Figure 6G. Thus, it appears that administering a precise dose of WEA can serve as a measure for protecting the intestinal tissue microstructure and mitigate the negative effects of LPS on tissue integrity. Then, we tested the levels of pro-inflammatory and Nrf2-related genes in mouse guts using qPCR. The transcription expression levels of IL-1β, IL-6, TNF-α, Nrf2, Keap1, and NQO1 in the the duodenum were tested. The IL-1β expression was significantly downregulated in only the WEA-L group (*p* < 0.05; Figure 6I), and the TNF-α expression was significantly downregulated in the WEA-L(*p* < 0.01), WEA-M (*p* < 0.05), and WEA-H (*p* < 0.05) groups (Figure 6J). On the other hand, WEA-L promoted the Nrf2 expression (*p* < 0.05; Figure 6K), and NAC (*p* < 0.05), WEA-L (*p* < 0.05), and WEA-H (*p* < 0.01) inhibited the duodenum Keap1 expression compared with the LPS group (Figure 6L). Collectively, our results indicate that WEA can alleviate inflammation and activate the Nrf2/Keap1 pathway in the gut by significantly reducing the mRNA expression of IL-1β and TNF-α.

### 3.6. The Alpha Diversity of Gut Microbiota

**Figure 7:**
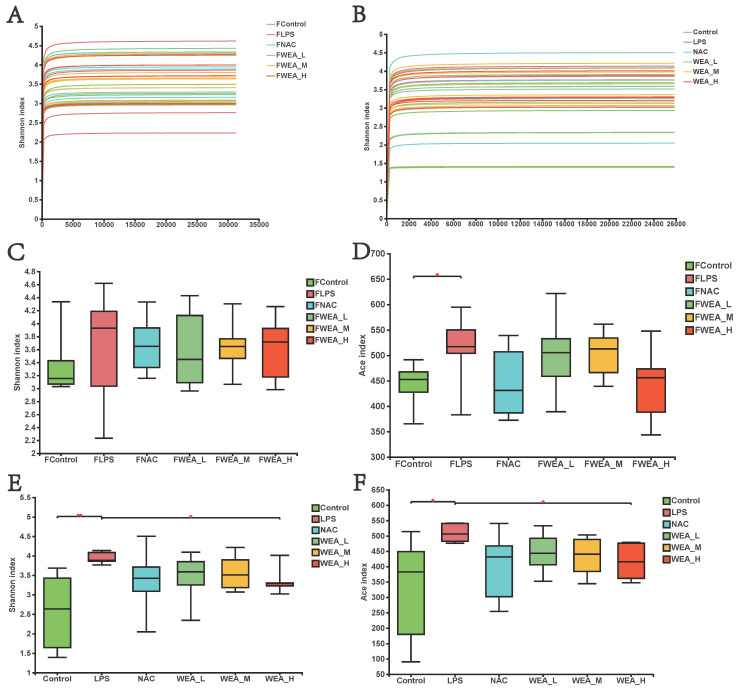
Gut microbiota alpha diversity. (**A**,**B**) Bacterial Shannon index curve, (**C**–**F**) alpha diversity analysis in the feces (**A**,**C**,**D**), and intestinal digesta (**B**,**E**,**F**). FLPS = 10 mg/kg lipopolysaccharide; FNAC = 300 mg/kg N-acetylcysteine; FWEA_L = 50 mg/kg water extract of *A. grossedentata;* FWEA_M = 100 mg/kg water extract of *A. grossedentata;* FWEA_H = 200 mg/kg water extract of *A. grossedentata;* LPS = 10 mg/kg lipopolysaccharide; NAC = 300 mg/kg N-acetylcysteine + 10 mg/kg lipopolysaccharide; WEA-L = 50 mg/kg water extract of *A. grossedentata* + 10 mg/kg lipopolysaccharide; WEA-M = 100 mg/kg water extract of *A. grossedentata* + 10 mg/kg lipopolysaccharide; WEA-H = 200 mg/kg water extract of *A. grossedentata* + 10 mg/kg lipopolysaccharide.

- The gut microbiome plays an essential role in the generation and development of oxidative stress. In this study, we examined whether the alleviation of inflammation by WEA in LPS-induced mice models is related to the modification of gut microbiology structure using 16S rRNA sequencing. As illustrated in Figure 7A,B, the flattened curve of the Shannon index suggests that the sequencing data obtained from the feces samples and intestinal digesta were adequate and trustworthy for use in further analysis. Subsequently, we evaluated the alpha diversity of all groups. WEA has no effect on the fecal flora of mice, and it is assumed that WEA is safe for mice (Figure 7C,D). However, the values for Shannon (*p* < 0.01; Figure 7E) and ACE (*p* < 0.05; Figure 7F) indices were significantly lower in the intestinal digesta in the control and WEA-H groups compared with the LPS group, indicating an ameliorative effect of WEA on the LPS-induced alterations in intestinal alpha diversity in mice.

### 3.7. Composition of Gut Microbiota

**Figure 8:**
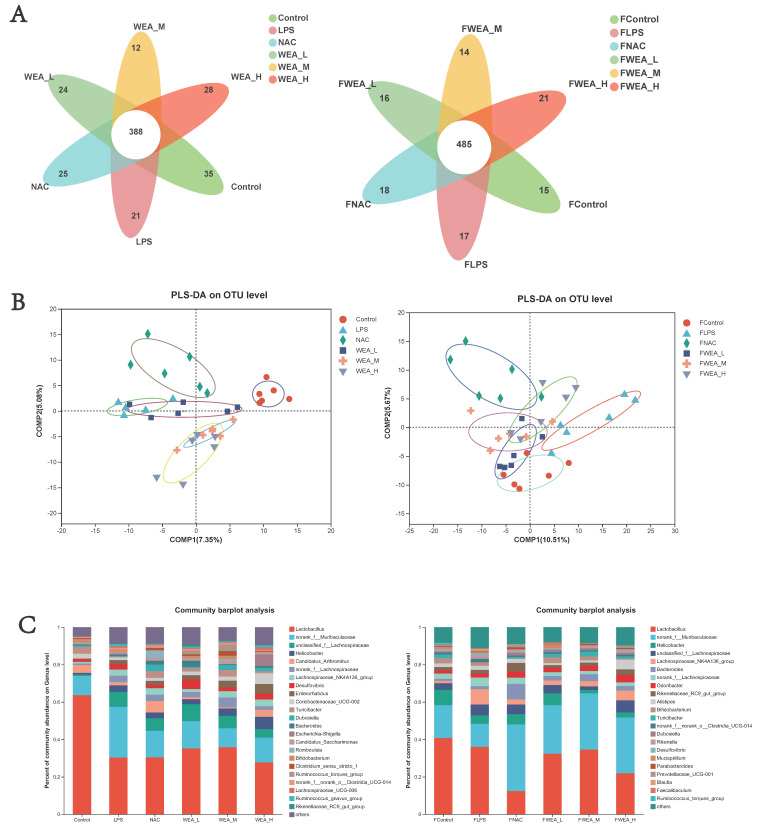
Composition of the gut microbiota. (**A**) Venn diagrams, (**B**) PLS-DA score plots, and (**C**) bacterial taxonomic compositions at the levels of genus in the intestinal digesta and the feces. FLPS = 10 mg/kg lipopolysaccharide; FNAC = 300 mg/kg N-acetylcysteine; FWEA_L = 50 mg/kg water extract of *A. grossedentata;* FWEA_M = 100 mg/kg water extract of *A. grossedentata;* FWEA_H = 200 mg/kg water extract of *A. grossedentata;* LPS = 10 mg/kg lipopolysaccharide; NAC = 300 mg/kg N-acetylcysteine + 10 mg/kg lipopolysaccharide; WEA-L = 50 mg/kg water extract of *A. grossedentata* + 10 mg/kg lipopolysaccharide; WEA-M = 100 mg/kg water extract of *A. grossedentata* + 10 mg/kg lipopolysaccharide; WEA-H = 200 mg/kg water extract of *A. grossedentata* + 10 mg/kg lipopolysaccharide.

- The Venn diagram shows the unique and shared gut OTUs of different groups in the feces and intestinal digesta. The numbers of LPS-induced unique OTUs in both feces and intestinal digesta decreased after WEA treatment (Figure 8A). To identify the potential effects of different doses of WEA on the LPS in mice, we incorporated PLS-DA to directly visualize the discrepancies in the microbiology profiles of the six groups. PLS-DA scoring plots showed that, after the mice were treated with different component fractions, there was a clear classification of microbiota composition across groups, with samples from the same group clustered together. In a sample of feces, the effect of WEA intervention on the microorganisms was similar to that of the control group, with a notable intersection between the low-dose WEA and control groups, the FLPS group was significantly different from the other groups, resulting in no intersection (Figure 8B). However, in a sample of intestinal digesta, medium and high doses of WEA were clustered together, but the low dose of WEA and LPS groups showed some intersection.
- An analysis of the phylum level of microbiota in the feces showed that Bacteroidetes and Firmicutes were the dominant phyla in all groups. The FLPS group showed a relative increase in Firmicutes (66%) and a relative decrease in Bacteroidetes (23%) compared to the control group (59% and 26%, respectively). In contrast, the FWEA-L, FWEA-M, and FWEA-H groups showed relative decreases in Firmicutes (52%, 50%, and 44%) and relative increases in Bacteroidetes (34%, 43%, and 48%). A similar trend was observed in the intestinal digesta, where Bacteroidetes and Firmicutes were the most prevalent phyla, representing a combined 80% of the total population. The LPS group showed a relative decrease in Firmicutes (56%) and a relative increase in Bacteroidetes (30%) compared with the control group. Conversely, the WEA-L and WEA-M groups exhibited a relative increase in Firmicutes (63% and 81%, respectively) and a relative decrease in Bacteroidetes (21% and 13%). This suggests that the WEA treatment may have had an impact on the relative abundances of these two prevalent phyla (Figure S1).
- The genus-level analysis of the microbiota in the feces (Figure 8C) revealed that *Lactobacillus* was the most abundant genus across all groups. However, when comparing the FLPS group with the control group, there is a decrease in the relative abundance of *Lactobacillus* in the FLPS group (36% compared with 41% in the control group). Additionally, the relative abundance of *Lactobacillus* was lower in the FWEA-L and FWEA-M groups compared with the FLPS group (32% and 35%, respectively). The FLPS group also had a lower relative abundance of *norank_f__Muribaculaceae* (12%) than the FWEA-L, FWEA-M, and FWEA-H groups (26%, 30%, and 30%, respectively). These findings suggest that the WEA treatment may have had a positive impact on the relative abundances of *Lactobacillus* and *norank_f__Muribaculaceae* in feces at the genus level, potentially implying a healthier gut microbiome.
- The results of this study demonstrate that the WEA treatment has a positive effect on the gut microbiome by maintaining the abundance of beneficial bacteria and decreasing the abundance of harmful bacteria in feces. The genus-level analysis of the microbiota present in the intestinal digesta (Figure 8C) reveals that *Lactobacillus* is the most prevalent genus in all groups. However, there was a relative decrease in the abundance of *Lactobacillus* in the LPS group (30%) compared with the control group (64%). In contrast, the WEA-L and WEA-M groups display a relative increase in the abundance of *Lactobacillus* (35% and 36%, respectively). Additionally, the LPS group shows a relative increase in the abundance of *norank_f__Muribaculaceae* (28%). On the other hand, after the administration of WEA-L, WEA-M, and WEA-H, we saw a relative decrease in the abundance of *norank_f__Muribaculaceae* (15%, 10%, and 12%, respectively). This suggests that the WEA treatment may impact on the relative abundance of *Lactobacillus* and *norank_f__Muribaculaceae* at the genus level in the intestinal digesta.
- The findings demonstrate that LPS led to alterations in the gut microbiota, as evidenced by changes in the relative abundances of different phyla at both the fecal and intestinal digesta levels. Furthermore, the results indicate that the WEA intervention had a specific impact on the gut microbiome at the phylum and genus levels. The changes observed in the relative abundances of *Bacteroidetes* and *Firmicutes* in the intestinal digesta suggest that WEA may selectively promote the growth of certain beneficial bacteria while inhibiting the growth of harmful bacteria. Additionally, the WEA treatment appears to have had a greater impact on the microbial community within the intestinal digesta than in the feces, further emphasizing the specific nature of its effects on the gut microbiome. These findings underscore the potential utility of WEA as a therapeutic strategy for restoring the gut microbial balance and improving intestinal health.

### 3.8. Specific Bacterial Taxa at the Genus Level among the Six Groups

**Figure 9:**
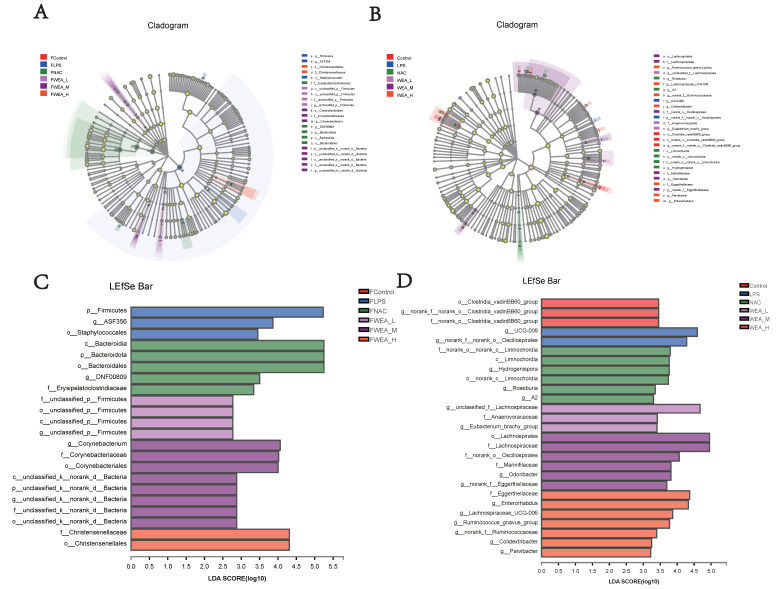
LEfSe analysis of the gut microbiota. Linear discriminant analysis of the gut microbiota in the (**A**,**C**) feces and the (**B**,**D**) intestinal digesta. (**A**,**B**) Evolutionary branch diagram. (**C**,**D**) LDA value distribution histogram, listing taxa meeting the LDA score threshold > 2. FLPS = 10 mg/kg lipopolysaccharide; FNAC = 300 mg/kg N-acetylcysteine; FWEA_L = 50 mg/kg water extract of *A. grossedentata;* FWEA_M = 100 mg/kg water extract of *A. grossedentata;* FWEA_H = 200 mg/kg water extract of *A. grossedentata;* LPS = 10 mg/kg lipopolysaccharide; NAC = 300 mg/kg N-acetylcysteine + 10 mg/kg lipopolysaccharide; WEA-L = 50 mg/kg water extract of *A. grossedentata* + 10 mg/kg lipopolysaccharide; WEA-M = 100 mg/kg water extract of *A. grossedentata* + 10 mg/kg lipopolysaccharide; WEA-H = 200 mg/kg water extract of *A. grossedentata* + 10 mg/kg lipopolysaccharide.

- The LDA value distribution histogram and an evolutionary branch diagram were constructed following the LDA effect size (LEfSe) analysis, and these can be used to discover biomarkers with statistical differences among experimental groups. The LEfSe taxonomic cladogram shows the key bacterial alterations, with different colors representing the different groups and sizes of circles indicating the relative abundance. The results reveal significant variations in the composition of gut microbiota among the different groups. In particular, the LEfSe analysis identified a number of genera that act as biomarkers for taxa with notable differences among the six groups. In the feces (Figure 9A,C), there were 22 genera that were identified as biomarkers, with *Firmicutes*, *ASF356,* and *o_Staphylococcale* being specific to the FLPS group. The LEfSe analysis revealed that the FLPS group had fewer differential biomarkers than the FWEA groups. Similarly, in the intestinal digesta (Figure 9B,D), there were 27 genera that were identified as biomarkers, with *Clostridia_vadinBB60_group, norank_f_norank_o_Clostridia_vadinBB60_group,* and *f_norank_o_Clostridia_vadinBB60_group* being dominant in the control group. In the LPS group, two key genera were identified as specific bacteria, namely *UCG-009* and *g_norank_f__norank_o_Oscillospirales*, which may have played a role in the pathogenesis of oxidative stress.

### 3.9. Correlation Analysis of Gut Microbiota and Inflammatory and Oxidative Factor Parameters

**Figure 10:**
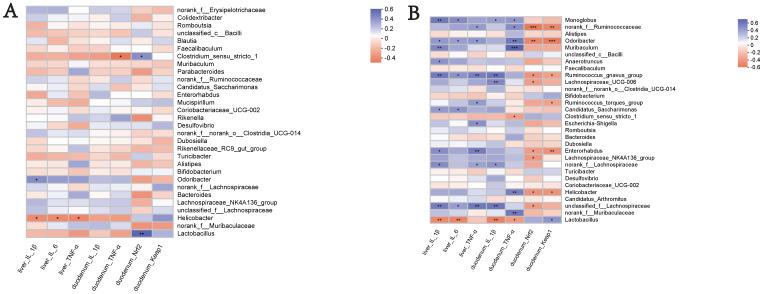
Key genera involved in mediating pro-inflammatory and antioxidant genes in the liver and duodenum: (**A**) fecal; and (**B**) intestinal digesta. Significant correlations are indicated in red for positive correlations and blue for negative correlations, while the independent color bars on the right depict correlation coefficients between the microbiota and genes. The correlation was considered significant when the absolute value of Spearman’s rank correlation coefficient was >0.6, and statistically significant when *p* < 0.05. The significance was as follows: * *p* < 0.05, ** *p* < 0.01, *** *p* < 0.001. IL-1β, interleukin 1β; IL-6, interleukin 6; TNF-α, tumor necrosis factor alpha; Nrf2, nuclear factor E2-related factor 2; Keap1, Kelch-like ECH-associated protein 1.

- An analysis was conducted to investigate the potential correlation between the gut microbiota at the genus level and the expression levels of specific genes, including IL-1β, IL-6, and TNF-α in the liver, and IL-1β and TNF-α in the duodenum, as well as Nrf2 and Keap1. This analysis was performed to gain a deeper understanding of the protective effects of the WEA administration on the gut microbiota and oxidative stress-related parameters in LPS-induced mice. The analysis of the genus-level results, described above, suggests that WEA treatment may have an effect on the relative abundance of *Lactobacillus* and *norank_f__Muribaculaceae* in the feces and intestinal digesta. Therefore, we focused on analyzing the correlation of *Lactobacillus* and *norank_f__Muribaculaceae* with specific genes.
- An analysis of the correlations in the fecal microbiota is depicted in Figure 10A. The expression of the specific genes examined was found not to be correlated with fecal microbiota (*Lactobacillus* and *norank_f__Muribaculaceae*) in mice, further demonstrating the safety of WEA. Furthermore, as shown in Figure 10B, an examination of the correlation between WEA intervention and intestinal microbiota revealed that the presence of the genus *Lactobacillus* is positively associated with Keap1 gene expression in the duodenum and negatively correlated with IL-1β and IL-6 expression in the liver as well as IL-1β and TNF-α expression in the duodenum. Furthermore, the abundance of *Lactobacillus* was found to be higher in the control and WEA groups. On the other hand, the genus *norank_f_Muribaculaceae* showed positive correlation with TNF-α expression in the duodenum, with lower abundance in the control and WEA groups. These findings suggest that the increase in *Lactobacillus* and decrease in *norank_f_Muribaculaceae* in the intestinal digesta resulting from WEA administration may contribute to the inhibition of inflammatory cytokine proliferation and the recovery of liver function. These results further demonstrate the safety and reliability of WEA and its effects on alleviating LPS-induced oxidative stress in mice.

### 3.10. Analysis of Drosophila Survival Rate

**Figure 11:**
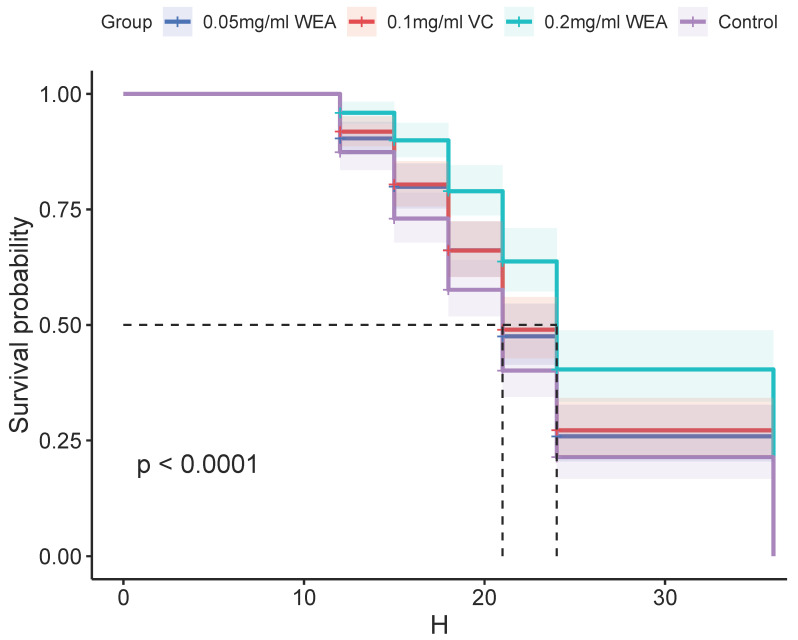
Survival curves of *Drosophila* reared on different diets: 0.05 mg/mL WEA = 0.05 mg/mL water extract of *A. grossedentata*+ 6 mM paraquat; 0.2 mg/mL WEA = 0.2 mg/mL water extract of *A. grossedentata* + 6 mM paraquat; 0.1 mg/mL VC = 0.1 mg/mL vitamin C + 6 mM paraquat.

- The antioxidant properties of WEA were further investigated using a *Drosophila* survival test. The results presented in Table 1 and Figure 11 demonstrate that WEA has the ability to increase the mean lifespan of *Drosophila* in a concentration-dependent manner, and the effects of 0.05 mg/mL WEA are similar to those of 0.1 mg/mL VC. Specifically, the groups treated with 0.05 and 0.2 mg/kg WEA showed a significant increase in the mean lifespan by 13.90% and 50.22%, respectively. Additionally, the maximum and median lifespans of the flies were also increased, with the most pronounced effects seen in the group receiving 0.2 mg/kg WEA. These findings indicate that WEA exhibits significant antioxidant properties as well as an ability to improve longevity in *Drosophila* by reducing oxidative stress. The results of this study further support the potential therapeutic benefits of WEA in a wide range of conditions associated with oxidative stress.

## 4. Discussion

In China, vine tea is a plant resource with both medicinal and edible properties, and it is known to possess a high flavonoid content [26]. One of its key flavonoid components is dihydromyricetin, which carries a range of biological functions. Previous research has shown a positive correlation between the flavonoid content and antioxidant effect [15,27]. However, the poor water solubility of DMY results in its low membrane permeability and bioavailability, limiting the widespread use of vine tea. As a result, in this study, the use of WEA was investigated as a means to enhance its antioxidant function and improve its bioavailability.

In this study, first, the cell assay results indicate that AEA and WEA, but not DMY, could reverse LPS-induced intestinal IPEC-J2 cell barrier dysfunction, with WEA demonstrating a more pronounced effect than AEA, which is in line with the findings of previous studies [28]. Additionally, the antioxidant activity of WEA was evaluated using four different in vitro assays, including DPPH, ABTS, FRAP, and reducing power. These assays are commonly used to measure the antioxidant activity. The results of this study are consistent with those of previous research [27]. Despite the positive results obtained through in vitro studies, it is important to note that the DPPH, ABTS, FRAP, and reducing power assays are limited models that do not take into account all of the antioxidant activities present in WEA. Furthermore, driven by these encouraging results, and given the diverse range of biological activities (such as antioxidant, anti-inflammatory, antitumor, antidiabetic, neuroprotective, and others) assigned to certain components of WEA, the further testing of WEA was conducted in vivo using mouse models [29].

Previous research has demonstrated that the flavonoid component DMY in WEA has the potential to ameliorate LPS-induced sickness and depressive-like behaviors in mice by inhibiting the TLR4/Akt/HIF1a/NLRP3 pathway [30]. Additionally, other studies have shown that the DMY has the ability to inhibit osteoclastogenesis and bone loss through scavenging LPS-induced oxidative stress and activating the NF-KB and MAPK pathways [31]. Furthermore, it has been discovered that vine tea, which contains WEA, can suppress the NF-kB signal pathway, thereby alleviating DSS-induced colitis [32]. Moreover, research has indicated that DMY can inhibit the expression of pro-inflammatory cytokines via activating the Nrf2 pathway in the RA model [33]. In the present study, we further investigated the molecular mechanism of WEA in relation to oxidative stress by establishing an inflammation model via LPS injection into the intraperitoneal area and then studying the effects of WEA. The results indicate that WEA administration significantly reduces the levels of IL-6, IL-1β, TNF-α, and T-AOC and increases the contents of CAT, which are markers of oxidative stress and inflammation used to indicate the presence of cellular damage.

The Nrf2/Keap1 signaling pathway plays a crucial role in the defense against oxidative and electrophilic stress, which can arise from both endogenous and exogenous sources [34]. The Keap1 protein mediates the ubiquitination and degradation of Nrf2 in both the cytoplasm and nucleus. Under normal physiological conditions, Keap1 binds to Nrf2 and targets it for proteasomal degradation [35], however, under conditions of oxidative stress, Nrf2 is released from Keap1 and translocates to the nucleus, where it binds to antioxidant response elements (AREs) or electrophile response elements (EpREs) to activate the transcription of downstream genes [36]. There is a growing body of evidence that suggests that various drugs can inhibit oxidative stress and inflammation by activating the Nrf2/Keap1 pathway. For instance, cardamonin, a natural flavone, has been shown to alleviate inflammatory bowel disease by inhibiting NLRP3 inflammasome activation through the Nrf2/NQO1 pathway [37]; chlorogenic acid, a polyphenolic, ameliorates oxidative stress and improves endothelial function in diabetic mice via Nrf2 activation [38]; pterostilbene suppresses oxidative stress and allergic airway inflammation through AMPK/Sirt1 and Nrf2/HO-1 pathways [39]. In the current study, we found that WEA administration significantly elevates the expression of Nrf2 and downstream antioxidants, such as NQO1, and decreases the expression of pro-inflammatory cytokines IL-1β and TNF-α resulting from LPS-induced oxidative stress in mice. Additionally, WEA effectively alleviates pathological damage to the liver and intestinal epithelium as well as increases the villus height and V/C in the duodenum, jejunum, and ileum. These effects may be due to the presence of flavonoids and DMY in WEA.

Increasing evidence suggests that oxidative stress and the gut microbiota are frequently linked [40,41,42]. Therefore, we explored the gut contents and fecal microbiota composition using 16S rRNA sequencing and evaluated oxidative stress-related intestinal bacterial indicators. In our study, in the gut contents, microbial growth differed between the different groups. In terms of alpha diversity, the LPS group presented significantly higher values of Shannon and ACE indices. According to the results, it appears that the presence of LPS has a discernible effect on the diversity and composition of the microbial population within the intestinal tract. This observation is consistent with a significant body of previous research that has also demonstrated this phenomenon [43,44,45,46,47]. LPS, which are commonly found in the cell wall of Gram-negative bacteria, have been shown to elicit significant alterations in the composition and structure of the gut microbiota. The dysregulation of gut microbial homeostasis due to LPS exposure can have significant impacts on the host’s physiology and health. This disruption in the microbial community’s balance can lead to the proliferation of pathogenic bacteria and the production of harmful metabolites. The resulting changes in the microbial composition and diversity can increase the permeability of the intestinal barrier, allowing the translocation of these noxious agents across the epithelial lining. This can trigger an inflammatory response and further damage the gastrointestinal system, exacerbating the impact on the host’s well-being. Therefore, maintaining a healthy gut microbial ecosystem is crucial for preserving the host health and preventing the onset of disease. In contrast, the WEA-H group showed converse results, indicating that oxidative stress and WEA significantly influence the diversity of the gut microbiota. Meanwhile, our results are in agreement with those of previous studies [17]. The PLS-DA analysis also indicated that, in all groups, the compositions of the gut microbiota were distinct, which further indicates that the gut microbiota reacts differently to different oxidative stresses.

In contrast, the WEA-H group showed converse results, indicating that oxidative stress and WEA significantly influence the diversity of gut microbiota. Meanwhile, our results are in agreement with those of previous studies [17]. The PLS-DA analysis also indicated that in all groups, the compositions of the gut microbiota were distinct, which further indicates that the gut microbiota reacts differently to different oxidative stresses.

In order to clarify how WEA reshapes the gut microbiota composition, comparisons of the relative abundances in differently treated groups were conducted. At the phylum level, the LPS group showed a lower abundance of Firmicutes and Bacteroidetes. In contrast, the WEA treatment was able to reshape the composition of the disordered gut microbiota. Traditionally, the F/B ratio, as the most important ratio in microbiome studies, may reflect the eubiosis or dysbiosis of the GI tract and is regarded as a representative parameter of health status [48]. However, in the present study, there was no significant difference in the F/B ratio between the groups. In addition, the abundance of *Lactobacillus* was higher and the abundance of *norank_f__Muribaculaceae* was lower in intestinal digesta following the WEA treatment. *Lactobacillus* has been recognized as a beneficial factor that can reduce intestinal toxins, control the growth of pathogens, and alleviate inflammation responses [49]. *Norank_f__Muribaculaceae* is thought to be associated with ecological imbalance [50]. Moreover, current research shows that *Lactobacillus* in the intestinal digesta is positively associated with the expression of the Keap1 gene in the duodenum, and negatively correlated with the expression of IL-1β and IL-6 in the liver, as well as the expression of IL-1β and TNF-α in the duodenum, and *norank_f_Muribaculaceae* in the intestinal digesta was positively correlated with the expression of TNF-α in the duodenum. However, in the correlation analysis, WEA was not found to have a significant impact on the fecal microbiota of the studied mice. This may be because the fecal samples of other groups, except for the LPS group, were collected before LPS injection. Therefore, we conclude that WEA has no negative effect on intestinal flora, and WEA is safe and reliable. The above results indicate that WEA may boost the growth of beneficial bacteria and inhibit the propagation of harmful bacteria, thus alleviating oxidative stress, which may have an inhibitory effect on the proliferation of inflammatory factors and the recovery of liver function.

According to the LEfSe analysis, different biomarkers in mice treated with WEA also confirmed that WEA can change the composition of gut microbiota, which showed varying oxidative stress responses. In this research, the gut microbiota in the WEA group presented higher abundances of beneficial bacteria and lower abundances of pathogenic bacteria. Additionally, in another study, LBLF therapy significantly restored the gut dysfunction brought on by a high-fat diet, altering the composition of the gut bacterial community by increasing the presence of beneficial microbiota and reducing harmful bacteria [51]. The results indicate that LPS disrupts the homeostasis of gut microbiota and WEA tends to limit the reproduction of pathogenic bacteria. Emerging evidence indicates that the flavonoid-rich *A. tenuissimum* flower could remedy glycolipid metabolic disorders and inflammation in diabetic mice by modulating protein expression and gut microbiota [52]. In another study, DMY was shown to reduce the hepatic lipid synthesis and inflammation through the modulations of gut microbiota [53].

*Drosophila melanogaster* (fruit fly) is an excellent model because of its short lifespan and the ease with which it can be grown. As a result, we finally investigated the antioxidant activity of WEA in *Drosophila*. The results are similar to those of the above experiments, indicating that WEA can increase the lifespan of *Drosophila*, thus further demonstrating its antioxidant properties.

Regrettably, our study also has some limitations. For example, the sample size is relatively small, and research on mechanisms (relevant experiments, Western blot analysis) was not carried out due to the lack of tissue samples. Although we analyzed WEA’s ability to alleviate oxidative stress and intestinal inflammation, the exact monomer that plays this role needs to be further identified. In addition, although we determined that gut microbiota may also be involved in the alleviation of oxidative stress, further in-depth studies with fecal transplantation are needed to confirm the regulatory role of microbiota. In future studies, all these factors will be considered and addressed.

## 5. Conclusions

In summary, our in vitro results indicate that WEA can reverse LPS-induced IPEC-J2 cell intestinal barrier dysfunction, and within a certain range, the mass concentration of WEA is directly proportional to the DPPH and ABTS radical scavenging rate as well as the FRAP and reducing power. We sought to further investigate the molecular mechanism of WEA’s action on oxidative stress using LPS-induced mice as a model. According to this study, WEA alleviates oxidative stress by regulating the Nrf2/Keap1 pathway, thereby suppressing the inflammatory response. In addition, WEA not only significantly impacts the diversity of gut microbiota but may also boost the growth of beneficial bacteria and inhibit the propagation of harmful bacteria. We assume that the beneficial effects of WEA on oxidative stress might be mediated by changes in the gut microbiota. On the other hand, WEA also exhibited strong antioxidant capacity in the *Drosophila* assays. The above results suggest that WEA is a promising option for remedying oxidative stress. However, the efficacy and safety of WEA are worth investigating in clinics in the future. Therefore, based on our results, a more in-depth study should be carried out to explore the consequences of altered gut microbiota, such as whether it results in alterations in the production of endogenous metabolites such as SCFA, enhanced gastrointestinal immunity, improved gut barrier function, inhibited development of inflammation, or whether it can prevent the occurrence of disease.

## Figures and Tables

**Table 1:** Effects of WEA on the lifespan of *Drosophila*.

Group	Mean Lifespan (Hours)	Median Lifespan (Hours)	Maximum Lifespan ^1^ (Hours)
Control	14.87 ± 0.07	21.00 ± 0.00	22.93 ± 0.93
0.1 mg/mL VC	17.67 ± 0.70	22.00 ± 1.00	23.60 ± 0.83
0.05 mg/kg WEA	16.93 ± 0.81	22.00 ± 1.00	23.87 ± 0.87
0.2 mg/kg WEA	22.33 ± 1.92 **	24.00 ± 0.00	27.20 ± 1.22 *

^1^ Maximum lifespan calculated as the mean lifespan of 10% of the longest-surviving *Drosophila* in each group. All values are expressed as mean ± SEM. Compared with the control group, * *p* < 0.05, ** *p* < 0.01.

## Data Availability

Data are contained within the article.

## Supplementary Materials

The following supporting information can be downloaded at: https://www.mdpi.com/article/10.3390/antiox12030547/s1, Figure S1: Bacterial taxonomic compositions at the levels of phylum in the feces and the intestinal digesta; Table S1: Primers used for real-time quantitative PCR for functional analyses. The datasets presented in this study can be found in online repositories. The names of the repository/repositories and accession number(s) can be found below: http://www.ncbi.nlm.nih.gov/ (accessed on 1 January 2023), PRJNA929682.
