# Supplementary material for: The Water Extract of Ampelopsis grossedentata Alleviates Oxidative Stress and Intestinal Inflammation
Source: Antioxidants (Basel). 2023 Feb 21;12(3):547. doi: 10.3390/antiox12030547 (PMC10045513; doi:10.3390/antiox12030547)
Supplement: Supplementary file 1 [file antioxidants-12-00547-s001.zip › antioxidants-2225043-supplementary.pdf]

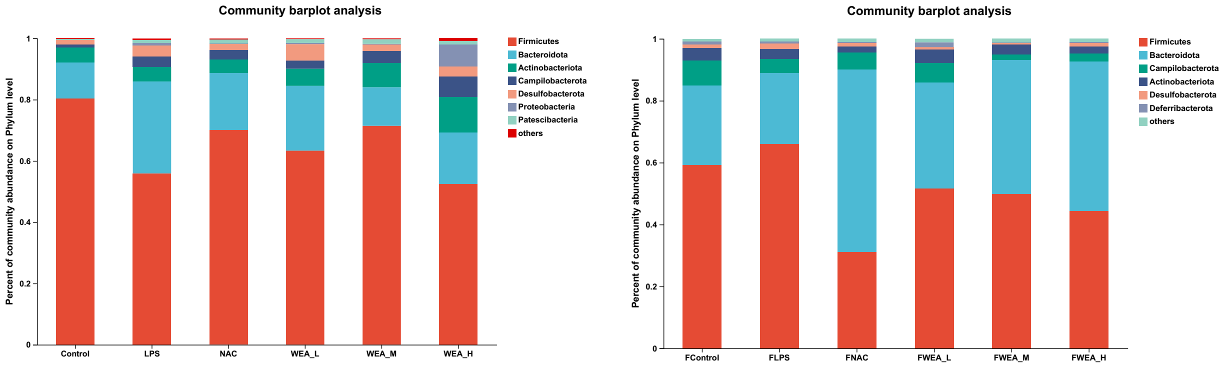

Figure S1: Bacterial taxonomic compositions at the levels of phylum in the feces and the intestinal digesta

Table S1: Primers used for real-time quantitative PCR for functional analyses

Genes	Forward primer	Reverse primer	Accession number
IL-6	CTCCCAACAGACCTGTCTATAC	CCATTGCACAACTCTTTTCTCA	XM_021163844.1
IL-1β	CACTACAGGCTCCGAGATGAACAAC	TGTCGTTGCTTGGTTCTCCTTGTAC	XM_006498795.5
TNF-α	ATGTCTCAGCCTCTTCTCATTC	GCTTGTCACTCGAATTTTGAGA	XM_021218154.1
GAPDH	CCTCGTCCCGTAGACAAAATG	TGAGGTCAATGAAGGGGTCGT	XM_029535298.1
Nrf2	CAGCCATGACTGATTTAAGCAG	CAGCTGCTTGTTTTCGGTATTA	XM_021193142.2
Keap1	GACTGGGTCAAATACGACTGC	GAGATGACTCGGAAGGATACTG	XM_032909789.1
NQO1	GAAGACATCATTCAACTACGCC	GAGATGACTCGGAAGGATACTG	NM_008706.5

Table S2: Chemical composition in WEA（TOP 10）

**Formula**	**Material**	**Secondary classification**
C21H20O12	Quercetin- -5-O-β-D- glucopyranoside	Flavonol
C15H12O7	Taxifolin (Dihydroquercetin)	Dihydroflavonol
C28H32O14	Acacetin-7-O- rutinoside (Buddleoside)	Flavone
C20H18O12	Myricetin-3-O- arabinosidechloride*	Flavonol
C20H18O11	Morin -3-O- xyloside	Flavonol
C21H20O12	Isohyperoside	Flavonol
C15H10O8	Quercetagetin	Flavonol
C22H24O9	3,5,6,7,8,3',4'- HeptaMethoxyflavone	Flavonol
C15H12O8	Dihydromyricetin (ampelopsin)	Dihydroflavonol
C21H24O10	dihydrochalcone -4'-O- glucopyranoside	Chalcone
